# Adhesion and Formation of Bacterial Biofilm on Polydioxanone (PDO) Thread Cannulas: An In Vitro Evaluation

**DOI:** 10.1111/jocd.70829

**Published:** 2026-04-01

**Authors:** Heloisa de Oliveira Rabelo, Letícia de Andrade Cunha, Heloisa Moreira Dias Pereira, Evelyn Priscilla Fregni da Silva Ryal, Lucas Guilherme Toshio Takeuti Wada, Emilly Brito Ferreira, Fabrícia Gimenes, Maria Cristina Bronharo Tognim

**Affiliations:** ^1^ Department of Basic Health Sciences State University of Maringá Maringá, Paraná Brazil; ^2^ Post‐Graduate Program in Biosciences and Physiopathology State University of Maringá Maringá, Paraná Brazil; ^3^ Post‐Graduate Program in Food Science State University of Maringá Maringá, Paraná Brazil

**Keywords:** adhesion, biofilm, cannulas, PDO‐TC

To the Editor,

Polydioxanone (PDO) threads are widely used in aesthetic procedures due to their *lifting effect* and collagen stimulation. For insertion, these threads are attached to a cannula that remains in contact with the patient's skin during the tissue defibrillation procedure to ensure correct insertion of the thread into the dermis [1]. Although minimally invasive, failures in biosafety practices can lead to complications, including infections. In such cases, bacteria may adhere to the thread or cannula, promoting biofilm formation and consequently hindering effective treatment [2]. Despite its clinical relevance, there is a scarcity of studies on the adhesion and formation of biofilms in PDO thread cannulas (PDO‐TC). Thus, the evaluation of these processes is essential to support preventive measures and improve the safety of aesthetic procedures.

Six standard strains (ATCC) were used: *Acinetobacter baumannii* (ATCC19606), *Staphylococcus aureus* (ATCC25923), *Staphylococcus epidermidis* (ATCC35984), *Enterococcus faecalis* (ATCC29212), *Escherichia coli* (ATCC25922), and *Pseudomonas aeruginosa* (ATCC27853). These species were selected due to their recognized ability to adhere and to form biofilms, coupled with the fact that many of these species colonize the skin surface of humans.

ProDEEP cannulas (Alur Medical, Novo Hamburgo, RS, Brazil), model MONO 30G‐38mm‐50 mm, were tested. The cannulas were cut into 1 cm fragments and incubated in 24‐well plates containing 1 mL of bacterial suspension at 0.5 on the McFarland scale at 37°C for 30 min, 2 h, 12 h, and 24 h. After each period, the fragments were washed with sterile saline solution and, after rinsing (removal of non‐adherent bacteria), they were transferred to a new tube with saline and vortexed for 2 min to release adherent bacteria. The suspensions were serially diluted to 10^−3^, seeded on Mueller‐Hinton Agar (20 μL, two drops per concentration), and incubated at 37°C for 24 h. Colony counting allowed quantification in Colony Forming Units/mL (CFU/mL). All tests were performed in duplicate. Adherence to PDO‐TC was also visually verified on semi‐solid agar (Figure 1).

**FIGURE 1:**
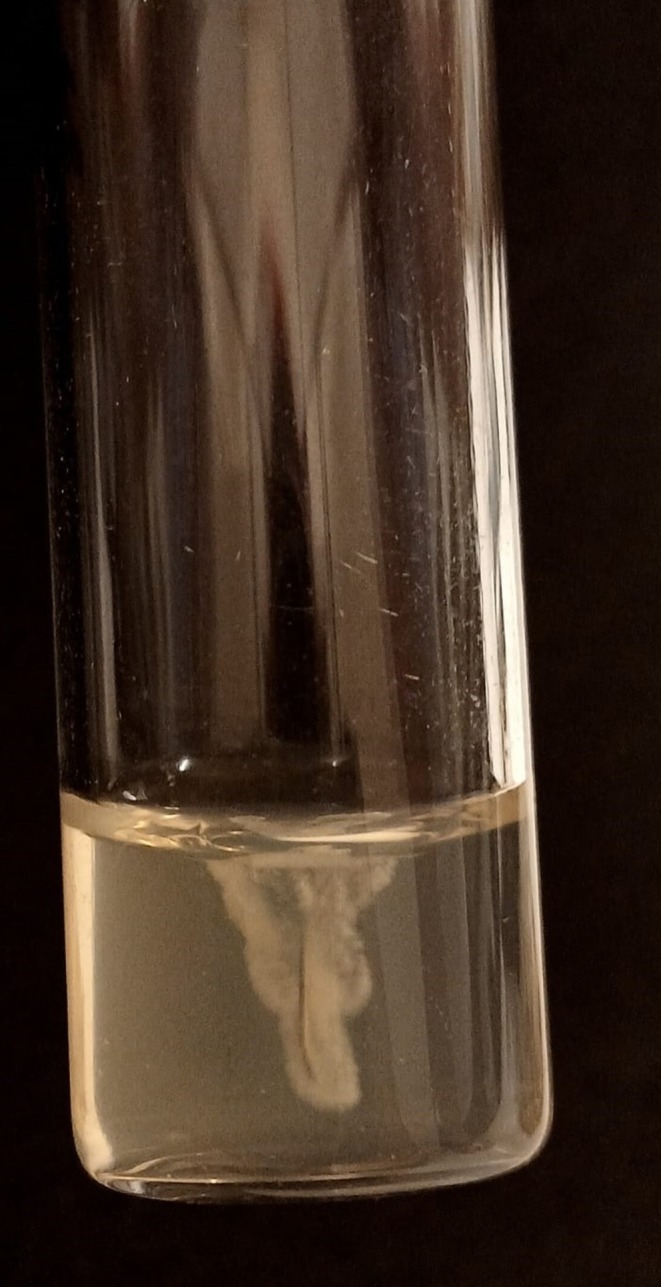
Visual verification of bacterial adherence on semi‐solid agar. Representative image of the assay used to evaluate the adhesion of bacterial to PDO‐TC. The distribution of bacterial growth around the material provides qualitative confirmation of the adhesion pattern described in the manuscript.

All strains adhered to PDO‐TC within the first 30 min, with a progressive increase over time. *P. aeruginosa* stood out with the highest number of adhered cells and upper limit of quantification (ULOQ) growth at all evaluated intervals. *S. aureus* showed the lowest adherence. After 12 and 24 h, an intensification of adherence was observed, especially for *A. baumannii* and *S. epidermidis* , which also reached ULOQ growth (Table 1).

**TABLE 1:** Quantitative analysis of bacterial adhesion in PDO‐TC (log_10_ CFU/mL).

Time	Quantification of adherence in CFU/mL					
	*A. baumannii*	*S. aureus*	*S. epidermidis*	*E. faecalis*	*E. coli*	*P. aeruginosa*
30 m	3.93	3.57	4.0	4.04	4.11	> ULOQ^a^
2 h	5.0	3.79	4.81	4.81	4.76	> ULOQ^a^
12 h	> ULOQ^a^	4.0	> ULOQ^a^	5.04	5.23	> ULOQ^a^
24 h	> ULOQ^a^	4.04	> ULOQ^a^	5.26	5.97	> ULOQ^a^

Abbreviations: CFU/mL: Colony Forming Units/mL; m: minutes; h: hours; ULOQ: upper limit of quantification.

^a^ ULOQ for our assay was 6.47 log_10_ CFU/mL, representing the maximum detectable bacterial adhesion under the established experimental conditions.

The high adherence of *P. aeruginosa* is associated with the expression of biofilm‐forming genes, which favors its persistence in hospitals. This ability, especially in catheters, tubes, and prostheses, contributes to persistent infections [3]. *S. aureus* and *S. epidermidis* showed progressive adherence over time; this more pronounced in *S. epidermidis* . Both are part of the cutaneous microbiota and can easily contaminate PDO‐TC during aesthetic procedures and cause infections [4].

*A. baumannii* exhibited strong adherence to PDO‐TC, corroborating its persistence in the hospital environment which, combined with its high antimicrobial resistance, can cause serious infections [3]. *E. coli* and *E. faecalis* also showed progressively increasing adherence, consistent with their ability to colonize medical devices [2].

Visual assessment on semi‐solid agar showed that, even after washing and intense agitation, the six strains remained adhered to PDO‐TC at all times. After initial fixation, adhesion can become strong in minutes, evolving until it may favor early biofilm development, which hinders the action of antimicrobials and increases microbial resistance [3, 5].

Preliminary data are relevant because they demonstrate, for the first time, the rapid bacterial adhesion to PDO‐TC, with variation between species and a progressive increase over time, which may favor early biofilm development. *P. aeruginosa* , *A. baumannii,* and *S. epidermidis* stood out for their high adhesion potential, highlighting a significant risk of contamination during aesthetic procedures. These findings reinforce the importance of rigorous biosafety and aseptic protocols in PDO‐TC insertion techniques to minimize the risk of infections associated with bacterial adhesion.

## Author Contributions

H.O.R., L.A.C., H.M.D.P., E.P.F.S.R., and L.G.T.T.W.: methodology, investigation, formal analysis, data curation. E.B.F.: formal analysis, writing – review and editing, writing – original draft. F.G. and M.C.B.T.: writing – review and editing, writing – original draft, visualization, validation, supervision, project administration, conceptualization.

## Funding

The authors have nothing to report.

## Ethics Statement

The authors have nothing to report.

## Consent

The authors have nothing to report.

## Conflicts of Interest

The authors declare no conflicts of interest.

## Data Availability

The data that support the findings of this study are available from the corresponding author upon reasonable request.
